# Does community care work? A model to evaluate the effectiveness of mental health services

**DOI:** 10.1186/1752-4458-2-10

**Published:** 2008-07-05

**Authors:** Emiliano Monzani, Arcadio Erlicher, Antonio Lora, Piergiorgio Lovaglio, Giorgio Vittadini

**Affiliations:** 1 Dipartimento di Salute Mentale dell'Azienda Ospedaliera "G. Salvini", Garbagnate Milanese, Italy; 2 Dipartimento di Salute Mentale dell'Azienda Ospedaliera Niguarda Cà Granda, Milano, Italy; 3 Dipartimento di Salute Mentale dell'Azienda Ospedaliera di Vimercate, Italy; 4 Dipartimento di Statistica dell'Università degli Studi di Milano Bicocca, Italy

## Abstract

The aim of this paper is to evaluate the effectiveness of community Mental Health Departments in Lombardy (Italy), and analyse the eventual differences in outcome produced by different packages of care. The survey was conducted in 2000 on 4,712 patients treated in ten Mental Health Departments. Patients were assessed at least twice in a year with HoNOS (Health of the Nation Outcome Scales). Data on treatment packages were drawn from the regional mental health information system, which includes all outpatient and day-care contacts, as well as general hospital and inpatient admissions provided by Mental Health Departments. Multilevel growth models were used for outcomes statistical analysis, expressed in terms of change of the total HoNOS score. On the whole, Mental Health Departments were effective in reducing HoNOS scores. The main predictor of improvement was treatment, while length of care, gender and diagnosis were weaker predictors. After severity adjustment, some packages of care proved more effective than others. Appropriate statistical methods, comprehensive treatment descriptions and routine outcome assessment tools are needed to evaluate the effectiveness of community mental health services in clinical settings.

## Background

Over the last 25 years a community care model for patients with mental disorders has been set up. This psychiatric care model is centred on a community-integrated network of mental health facilities (Community Mental Health Centres (CMHC), Psychiatric Wards in General Hospitals, Residential Facilities and Day-care Facilities) located in the neighbourhood, and coordinated by the Mental Health Department (MHD); this model has nothing to do with mental hospitals.

Despite this model's dissemination throughout Italy and other European and non-European countries, its effectiveness has never been properly assessed. Indeed, there have been few analyses of the system taken as a whole, although there are many assessments of the individual facilities and activities. Comparisons have been made of innovative and routine services, e.g. the PRISM "psychosis study" [1], and local area studies such as the South Verona Outcome project [2], but data to analyse the effectiveness of the daily practice of community mental health services on a large geographic scale are still scarce. An exception to this can be seen in the Australian Mental Health National Outcomes and Casemix Collection (MH-NOCC), in which Burgess et al. [3] describe care episodes provided for adults in public mental health services across Australia. Score changes and effect size on Health of the Nation Outcome Scales (HoNOS) [4] were calculated for 14,659 acute inpatient episodes and 23,692 community episodes. The results showed a general improvement for people in contact with the public sector of mental health services, though the level of change depended on the setting and type of episode. In the present analysis the episodes of care effectiveness were analyzed separately according to episode type.

In the year 2000, the psychiatric network in Lombardy consisted of 63 public Departments of Mental Health, 16 officially licensed private community residential facilities, 4 officially licensed private Day Centres and 3 private psychiatric clinics. Each public Department of Mental Health had, on average, a Psychiatric Ward with 15 beds in a General Hospital, 2 Community Mental Health Centres, 2 Community Residential Facilities and 1 Day Care Centre.

Let us look a moment at the present situation for a population of 10,000 people over the age of 15: The psychiatric facilities network consists of 96 Community Mental Health Centres, 68 Day Care Centres (1.3 daily attendance at each), 58 Psychiatric Wards in General Hospitals (1 bed for each) and 176 Community Residential Facilities (2.3 beds for each). In addition there are 3 active private Psychiatric Clinics (0.2 bed in each).

Today Mental Hospitals, as such, no longer exist in Italy; the last ones, which, during the '80s and '90s had wards for long term patients, were closed in 1999. At that time the Lombardy population aged over 15 years was 7,926,581.

In any case, difficulties can arise when trying to make an adequate evaluation of the effectiveness of mental health services, and these concern:

1. The evaluation of a patient's clinical and psycho-social problems during daily living. Several routine outcome assessment tools are now available, but to be acceptable such tools must be effective – this means they must be reproducible, easy to use (by involved professionals), sensitive to change, able to cover clinical and psychosocial issues and, finally, usable in different settings [5].

2. Description of treatment. Community Mental Health Centres (CMHCs) dispense different treatments, and this information must be summarized in order to evaluate the overall effectiveness of the services. Indeed, the treatment package is an interesting analysis model as it overrules artificial separations in the analysis of mental health activities, and focuses on the complexity of the mental health system. According to the UK NHS definition [6], a package of care is "a cluster of services provided to an individual based on carefully constructed components". Thus, such a package includes the characteristics of the patient, the type of treatment and the intensity of the care provided. Packages of care can be summed up as 'the mix of treatments provided to an individual patient within a specific timeframe involving different settings' e.g., CMHCs, day-care facilities, general hospital wards, community residential facilities) [7].

3. Statistical analysis of data. The most promising instruments for the evaluation of clinical services effectiveness are longitudinal models, and this is due to the recent improvements that have been made to such models [8]. The data that lead to the inclusion of a patient in a specific group are derived by individual decisions, not acquired through random patient allocation, thus the selection process itself can be the source of outcome differences. In order to limit such bias, any statistical analysis of effectiveness must meet the "*coeteris paribus*" rule with respect to the multiple treatment-provider principle; this means that the efficacy indicator must be independent of other operator-dependent confounding factors like differences in patient severity at the beginning of treatment. Therefore, comparisons need to be adjusted for severity, or case-mix. Risk adjustment is a statistical control process of the characteristics of patients that participate in studies concerning treatment quality, costs and outcome [9]. It has been proven that comparisons of severity-adjusted data and unadjusted data produce results different from those made of treatment and/or setting performance [10].

The aim of the present study was to evaluate the effectiveness of community Mental Health Departments in Lombardy, applying the above described methodological innovations, and particularly by analysing:

1) care package appropriateness;

2) MHD overall effectiveness

3) effectiveness of the different care packages.

## Methods

### A. Instruments

#### a. HoNOS

HoNOS was developed by a Research Unit [4] of the UK Royal College of Psychiatrists to routinely measure consumer outcome in mental health services. It is a tool for clinicians, consisting of 12 five-point scales from 0 (no problem) to 4 (severe/very severe problem), and covers clinical and psychosocial problems (8 clinical items, 4 psychosocial). As a tool it is useful and sensitive, articulately describing the current severity of a patient's condition, not only clinically but also in behavioural and psychosocial areas. HoNOS should be used whenever there is a need for a detailed characterisation of clinical and social problems. In a recent analysis involving the comparison of 4 routine measures, including HoNOS and GAF, the best coverage of patient problems was that of HoNOS [11]. Furthermore, assessment is rapid as HoNOS is easily and quickly filled-out by psychiatrists, psychologists and other professionals. It has been officially adopted as an outcome tool in the United Kingdom, Australia, and New Zealand, and is widely employed in surveys in many European Countries; during HoNOS 2 Research in Italy the program was translated and validated [12].

#### b. Care Packages

The data of care packages were derived from the regional mental health information system, thus providing a "psychiatric case register" as the system collects together demographic information concerning the patients themselves, their diagnostic characteristics and their contact with facilities. Such an information system gathers data from all the public Mental Health Departments as well as from private Day-care and Residential Facilities, allowing a full description of the epidemiological scenario and the monitoring of services provided to patients. This information system fits, at least partially, the criteria recommended by Rosembeck et al. [13] to implement a database able to provide solid and meaningful results.

The Care Packages were arranged in two steps:

a) CMHC contact, day-care attendance, days spent in psychiatric wards in General Hospitals and Community Residential Facilities were linked to each patient for the 1/1/2000 – 31/12/2000 period.

b) Six packages of care were identified, following a scheme presented in a previous analysis (Lora et al., 2002). The Care Packages derive from possible combinations of four different settings (CMHCs, Day-care facilities, Psychiatric Wards in General Hospitals, Community Residential Facilities):

**1. Clinical **(CLIN) packages: patients treated over the survey year only in CMHC, by psychiatrists or psychologists.

**2. Community **(COMM) packages: patients treated only in CMHC but, in addition to the psychiatrist/psychologist clinical programs, there was intervention by other professionals (such as nurses, social workers, rehabilitation therapists)

**3. Community – Day-Care **(COMM-DC) packages: in addition to CMHC activities the patients also attended Day-Care Centres;

**4. Community – Hospital **(COMM-HOSP) packages: in addition to CMHC activities the patients were also admitted to Psychiatric Wards in General Hospitals

**5. Community – Hospital – Day-care **(COMM-HOSP-DC) packages: the patients underwent treatment in CMHC, Day-Care Centres and in Psychiatric Wards in General Hospitals.

**6. Residential **(RES) packages: in addition to treatment in MHD facilities, the patients were admitted to a Residential Facility. Table 1

**Table 1:** Care Packages

		N	%
Clinical	CLIN	599	12.7%
Community	COMM	2784	59.1%
Community – Day-Care	COMM-DC	388	8.2%
Community – Hospital	COMM-HOSP	575	12.2%
Community – Hospital – Day-care	COMM-DC-HOSP	123	2.6%
Residential	RES	243	5.1%

#### c. Statistical model

A multilevel growth model [14,15], the longitudinal random effect model, was used for the statistical analysis. In studies that involve stratified groups, as is commonly found in health service research [16], such a model tends to improve the analysis of change: multilevel models are a primary *coeteris paribus *comparison tool to evaluate effectiveness, and thus represent the most commonly used medical research models [17]. Thus, multilevel models were developed for the statistical analysis of data in hierarchical structures, or in clusters, and were initially used in institutions like Departments of Education. They were later applied to evaluating other public utility services such as medical settings [18] and, particularly, mental health services [19].

When producing risk-adjusted data, one crucial factor is the choice of variables to analyze. In accordance with the modified Dow model [20], the current study considered, along with HoNOS scores, the following variables:

• Length of care

• Package of care;

• Diagnosis;

• Job;

• Marital status;

• Education level;

• Age;

• Occupation.

The statistical analysis was made using SAS software [21-23].

### B. Study design

The sample was extracted from a "HoNOS 2" study, conducted in 10 Mental Health Departments in Lombardy from the 1^st ^January 2000 to 31^st ^December 2000 to evaluate the relationship between the severity of disease and the cost of treatment [24]. The Mental Health Departments were the main providers of psychiatric services in an area with a population of 1,500,015, representing approximately 16% of the entire Lombardy population aged over 14 years.

The "HoNOS 2" study recruited 9,817 patients who had contact with Mental Health Departments three times during 2000: January (1^st ^assessment), June (2^nd ^assessment) and December (3^rd ^assessment). Not all the recruited patients were evaluated 3 times; 4,712 patients were evaluated at least twice (1^st^-2^nd ^assessment or 2^nd^-3^rd ^or 1^st^-3^rd ^or 1^st^-2^nd^-3^rd^) and only these were included in the analysis. Data missing from records did not in any way pose a limit to the survey as the employed statistical method overcomes this situation.

With regard to study design in mental health services, Lambert et al. [25] suggested the adoption of experimental models with three or more repeated measures, analyzed using multilevel longitudinal tools to avoid the criticism of a "one shot" outcome evaluation. Indeed, such analysis tools do not exclude those patients with only two evaluations or missing data from the study; in fact, missing data strengthens the statistical method.

Table 2 shows, for the 4,712 patients, the socio-demographical characteristics, diagnostic profile and previous contact with mental health services.

**Table 2:** Characteristics of the patients


Gender	Male	2073	44,0%

Age group	15/24 years	222	4,7%
	25/34 years	992	21,1%
	35/44 years	1127	23,9%
	45/54 years	994	21,1%
	55/64 years	805	17,1%
	more than 65 years	562	11,9%
	missing	10	0,2%

Marital status	single	2352	49,9%
	married	1646	34,9%
	separated – divorced	413	8,9%
	widow	246	5,2%
	missing	55	1,2%

Education level	primary school	1533	32,5%
	secondary school	1918	40,7%
	high school – university	1082	23,0%
	missing	179	3,8%

Living situation	alone	703	15,3%
	with parents	1858	38,6%
	with partner	1775	38,7%
	with other relatives	170	3,6%
	other living situation	125	2,0%
	missing	81	1,8%

Employement	not employed	896	67,0%
	missing	149	3,3%

Duration of contact with psychiatric services	less than 1 year	308	6,5%
	1 – 2	920	19,3%
	3 – 5	853	17,9%
	6 – 15	1674	35.7%
	> 15	923	20,0%
	missing	34	0,7%

ICD-10 diagnostic groups	Organic, including symptomatic, mental disorders	59	1,3%
	Mental and behavioural disorders due to psychoactive substance use	38	0,8%
	Schizophrenia, schizotypal and delusional disorders	2279	48,4%
	Mood disorders	971	20,6%
	Neurotic, stress-related and somatoform disorders	655	13,9%
	Behavioural syndromes associated with physiological disturbances and physical factors	57	1,2%
	Disorders of adult personality and behaviour	521	11,1%
	Mental retardation	113	2,4%
	Disorders of psychological development	3	0,1%
	Behavioural and emotional disorders with onset usually occurring in childhood and adolescence	16	0,3%

## Results

### 1) Care package appropriateness

According to the appropriateness criterion (higher HoNOS score, higher severity), the more severe the illness the more complex the treatment, severe conditions involving two or more types of services and the skills of several professionals. Our study revealed that appropriate treatment seems to be provided by the Lombard MHDs: On analyzing the initial total HoNOS score with the longitudinal adjusted model (Table 3), patients with a low mean severity score were treated with the CLIN package, involving only one professional operator (psychiatrist or psychologist) and one facility (CMHC); intermediate severity-scoring patients were given the COMM package, involving several professionals, or the COMM-DC package, involving CMHCs and Day-Care Centres, while patients with higher severity scores were given the packages involving more complex treatments and several facilities. Table 3

**Table 3:** Baseline level of HoNOS score for care packages

	"adjusted" baseline	p-value
CLIN	7,0	<.0001
COMM-DC	7,8	<.0001
COMM	7,9	<.0001
MEAN	9,1	<.0001
COMM-HOSP	10,4	<.0001
RES	11,2	<.0001
COMM-DC-HOSP	11,4	<.0001

### 2) Overall MHD effectiveness, and effectiveness predictors

The main outcome was the investigation into the reduction of the HoNOS score over time. Considering a multilevel growth approach, the model shows that the repeated HoNOS scores are strongly correlated, and variance is homogeneous over time.

Mental Health Department activity is effective in reducing the behavioural, clinical and psychosocial problems of treated patients, as shown by the significantly reduced HoNOS scores after treatment (Care Package).

More specifically, for all the packages, the HoNOS score over a 6-month assessment period showed a significant average reduction (0.45 points). Also to note is the significant decrease (0.9 points) in growth, or improvement rate, of the HoNOS score per year. (Table 4)

**Table 4:** Six-monthly improvement rate of honos score for care packages

	growth rate	p-value
COMM-HOSP	0,6166	<.0001
COMM-DC	0,6142	<.0001
COMM	0,5194	<.0001
MEAN	0,4501	<.0001
RES	0,4315	0,0011
COMM-DC-HOSP	0,2322	0,5347
CLIN	0,2184	0,0015

Given the overall effectiveness of the MHDs it can be seen that, with regard to outcome, the most influential variables are treatment type and length of care, thus they are the main improvement predictors.

Diagnosis and gender are less relevant, while social demographical variables (employment, marital status, education level, age) are the weakest predictors. No other variables proved statistically significant.

### 3) Effectiveness of the different care packages

Table 5 shows that the HoNOS score growth rate over time differs only marginally among the care packages (p = 0.071), nevertheless the differences in the improvement rates of the HoNOS scores over time for the different packages were investigated more deeply.

**Table 5:** Significant covariates for predicting HoNOS scores over time

	F Value	P
Time	40.45	<.0001
Care Package	79.28	<.0001
Time* of Care Package	1.72	0.0710
Diagnosis	22.81	<.0001
Gender	17.71	<.0001
Occupation	9.23	<.0001
Marital status	7.71	<.0001
Schooling	4.87	<.0001
First admission or contact with the facility	2.51	0.0198
Type of occupation	2.91	0.0125

**Figure 1:**
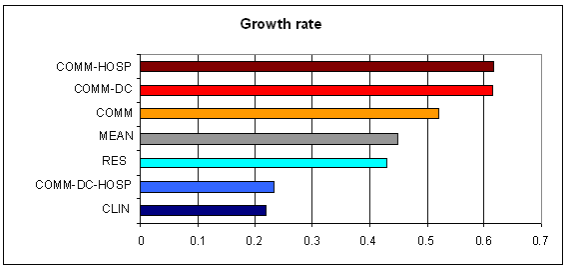
**Six-monthly improvement rate of honos score in relation to care package**. COMM-HOSP = Community – Hospital; COMM-DC = Community – Day-Care; COMM = Community; RES = Residential; COMM-DC-HOSP = Community – Hospital – Day-care; CLIN = Clinical.

Figure 1 depicts the adjusted HoNOS score at three time instants, showing that the greatest HoNOS score reduction occurred with the COMM-HOSP, COMM-DC and COMM packages, while the other three packages (RES, COMM-DC-HOSP and CLIN) were lower, i.e., a below-average score reduction. It can be seen from Table 5 that the COMM-DC-HOSP care package does not significantly reduce the HoNOS score over time.

**Figure 2:**
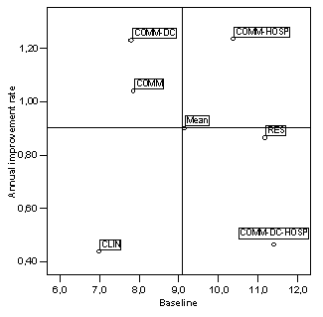
**Annual improvement rate and baseline severity**. COMM-HOSP = Community – Hospital; COMM-DC = Community – Day-Care; COMM = Community; RES = Residential; COMM-DC-HOSP = Community – Hospital – Day-care; CLIN = Clinical.

A joint analysis of severity (adjusted HoNOS score at baseline level) and improvement (annual rate of growth) for each care package was used to compare the effectiveness of the different packages. Figure 2 with its axes representing the patients' severity and annual improvement rate shows that the severity level and improvement rate are, for the CLIN package, below-average; for the COMM-DC-HOSP and RES packages, above-average and below-average; for the COMM and COMM-DC packages, below-average and above-average; while the COMM-HOSP package includes patients with above-average severity scores and improvement rates.

**Figure 3:**
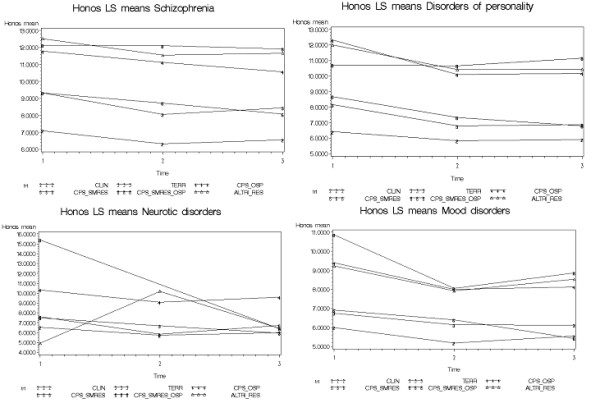
**Packages of care effectiveness in relation to diagnosis**. Figures show that care packages effectiveness can change in relation to the diagnosis.

Care package effectiveness varies according to the diagnosis (Table 5). The COMM package is effective for all diagnoses, the COMM-DC and COMM-HOSP packages for schizophrenia, affective disorders and personality disorders, the RES packages for schizophrenia and the COMM-DC-HOSP packages only for neuroses. (Figure 3 and table 6)

**Table 6:** Care effectiveness in relation to diagnosis

**DIAGNOSIS**	**TREATMENT**	**ESTIMATE**	**STDERR**	**TVALUE**	**PROBT**
**Schizophrenia**	CLIN	-0.26	0.21	-1.21	0.2257
	COMM	-0.42	0.07	-5.79	0.0000
	COMM-DC	-0.61	0.15	-3.98	0.0001
	COMM-HOSP	-0.63	0.15	-4.10	0.0000
	COMM-DC-HOSP	-0.12	0.29	-0.43	0.6651
	RES	-0.40	0.18	-2.22	0.0264
**Mood Disorders**	CLIN	-0.24	0.20	-1.24	0.216786
	COMM	-0.32	0.11	-2.94	0.003408
	COMM-DC	-0.58	0.21	-2.73	0.006415
	COMM-HOSP	-0.73	0.36	-2.03	0.042416
	COMM-DC-HOSP	-0.98	0.64	-1.54	0.123957
	RES	-0.37	0.42	-0.90	0.369404
**Neurotic Dis.**	CLIN	-0.28	0.16	-1.75	0.08151
	COMM	-0.77	0.09	-8.11	0.00000
	COMM-DC	-0.29	0.41	-0.72	0.47346
	COMM-HOSP	-0.39	0.35	-1.12	0.26549
	COMM-DC-HOSP	-4.50	1.61	-2.79	0.00551
	RES	0.35	1.99	0.18	0.86027
**Personality Dis.**	CLIN	-0.23	0.33	-0.69	0.49285
	COMM	-0.65	0.16	-3.93	0.00010
	COMM-DC	-1.10	0.34	-3.18	0.00157
	COMM-HOSP	-0.97	0.40	-2.42	0.01612
	COMM-DC-HOSP	0.22	0.49	0.46	0.64847
	RES	-0.74	0.65	-1.13	0.26031

## Conclusion

This study at MHDs in Lombardy has enabled a first-time evaluation of mental health service effectiveness in the region. However the results must still be considered preliminary, not only because the percentage of treated patients in the sample was small (less than 1/20), but also because the study was subject to some limitations at the methodological level. Thus, a different study design is needed: a cohort of patients should be followed and assessed, not only at six month intervals but also when settings change e.g., when a care episode concludes at one facility and treatment begins at another. Assessing patients after there has been a change in their settings allows a more comprehensive understanding of any severity changes that might occur. Further improvement to the model could be achieved by including intensity of care as a variable. Although this analysis did not evaluate care intensity, i.e. the number of community contacts, daycare centre attendance and days spent in hospitals and residential facilities provided within the packages, the splitting of each package into terms of high-, medium- and low- resource sub-packages, according to intensity, could refine the model.

Despite these limitations, the present study suggests that the network of community Mental Health Departments in Lombardy affects patient outcome positively; moreover, the organization of the study treatment blocks is appropriate as the most serious patients receive the most complex treatments.

Some packages are more effective than others in improving patient outcomes. Indeed, the package involving both hospital and community facilities gives the best results, while the package involving hospital, community and day-care activities turns out to be less effective. The limited or nil effect shown by the more complex packages, which involve several facilities (like the package with several residential facilities and the one including hospital, community and day-care activities), may be explained by the many difficulties encountered in the coordination and clinical governance of such excessively complex cases (in terms of facilities). Moreover this package is more likely to be reserved for more serious cases, patients who are "resistant to treatment" and not responsive to other, previously provided, less complex packages.

The reduced effectiveness of the clinical package is probably related to patient selection, according to the design of this study. A cross-sectional sample with repeated measurements such as this one tends to recruit chronic cases rather than onsets. Thus this selection may have produced a bias toward more complex packages rather than toward "lighter" treatments that, in fact, are often limited to a short ambulatory treatment; clearly the outcome of these treatments is not highlighted by this study design.

The results related to the effectiveness of community care, for patients with different diagnoses, are contradictory. On the one hand, the model is able to discriminate between the effectiveness of the different packages used for the same diagnosis just as the diagnosis can suggest the care package to be used, on the other hand, for example, the lack of CLIN package efficacy in neuroses is questionable. Thus it is quite clear that for a more conclusive evaluation, further research is needed into the relationship between care packages and diagnoses or, better still, between care packages and specific patient problems.

This survey represents the first evaluation of the effectiveness of community care in Italy involving a relatively large sample of patients. It confirms that it is possible to evaluate the effectiveness of community Mental Health Departments and that such evaluation can be conducted at reasonable costs, provided appropriate tools are used.

The feasibility of HoNOS is a crucial requirement for routine outcome assessments: indeed, it is unthinkable to have the use of more tools for a survey involving 10 Departments of Mental Health and 10,000 patients for a period of a year. Not only has HoNOS been proved useable, it has also been revealed to be "sensitive to change", and able to give a detailed picture of outcomes.

The information system that collects psychiatric information in Lombardy enabled both the sample recruitment and the complete elaboration of the data collection. Thanks to this system, it has been possible to accurately describe the care packages.

The used statistical method proved to be a suitable methodological instrument for an effective evaluation of growing and changing data, as highlighted by Gilbody [26] in his systematic review of mental health services outcomes. In fact, the multilevel growth models [14] not only protected the principle of "severity adjustment" but also brought several benefits, compared to traditional longitudinal models based on uni- or multivariate analysis of variance, contrast analysis and fixed effect models. By clearing the outcome of the effects due to individual differences and the different resources used, the models allow an analysis of context and case-mix variables at different levels of hierarchy, even in observational studies. Such models are flexible and applicable to normally distributed and non-normally distributed continuous outcome categorical variables. They are robust also in the case of irregular, dispersed, and missing data, and can contain time-invariant or time-variant covariates. Such variables often occur in administrative registries. The flexibility of these statistical methods allows the use of otherwise useless data, and enables the recruitment of larger samples.

We firmly believe that the combination of appropriate instruments for the evaluation of routine outcomes, patient-oriented informative systems and appropriate statistical methods will, in the future, lead to useful results that can be used to evaluate the effectiveness of community mental health care.

## Competing interests

The authors declare that they have no competing interests.

## Authors' contributions

All authors participated in the design of the study. EM, AL and PL wrote the manuscript. AE and GV reviewed the manuscript. PL and GV performed the statistical analysis. All authors read and approved the final manuscript.
